# Effective Photoluminescence Imaging of Bubbles in hBN-Encapsulated WSe_2_ Monolayer

**DOI:** 10.3390/nano10020350

**Published:** 2020-02-18

**Authors:** Seong-Yeon Lee, Tae-Young Jeong, Seonghun Ahn, Suyong Jung, Yong-Hoon Cho, Ki-Ju Yee

**Affiliations:** 1 Department of Physics, Chungnam National University, Daejeon 34134, Korea; lsy929292@naver.com (S.-Y.L.); feteri88@gmail.com (T.-Y.J.); 2 Korea Research Institute of Standards and Science, Daejeon 34113, Korea; syjung@gmail.com; 3 Department of Physics and KI for the NanoCentury, Korea Advanced Institute of Science and Technology (KAIST), Daejeon 34141, Korea; insutine12@kaist.ac.kr (S.A.); yhc@kaist.ac.kr (Y.-H.C.)

**Keywords:** 2D materials, hBN-encapsulated WSe_2_ monolayer, photoluminescence mapping, photoluminescence excitation spectroscopy

## Abstract

Interfacial bubbles are unintentionally created during the transfer of atomically thin 2D layers, a required process in the fabrication of van der Waals heterostructures. By encapsulating a WSe_2_ monolayer in hBN, we study the differing photoluminescence (PL) properties of the structure resulting from bubble formation. Based on the differentiated absorption probabilities at the bubbles compared to the pristine areas, we demonstrate that the visibility of the bubbles in PL mapping is enhanced when the photoexcitation wavelength lies between the n = 1 and n = 2 resonances of the A-exciton. An appropriate choice of detection window, which includes localized exciton emission but excludes free exciton emission, further improves bubble imaging capability. The interfacial position dependence of the bubbles, whether they are located above or below the WSe_2_ monolayer, gives rise to measurable consequences in the PL shape.

## 1. Introduction

Monolayers of transition metal dichalcogenides (TMDs) such as WSe_2_, WS_2_, MoSe_2_, and MoS_2_ have attracted considerable attention owing to their intriguing optical and electrical properties and possible applications in optoelectronic devices [1,2,3]. Strong Coulomb interaction in TMD monolayers (MLs) enables the investigation of excitonic complexes such as excitons, trions, and biexcitons [4,5,6,7]. Recent research on TMD MLs has focused on heterostructures comprising different types of 2D materials with the purpose of finding new physical properties and high sample quality [8,9]. For instance, by encapsulating a TMD ML with hBN layers on both bottom and top regions, an exciton linewidth as narrow as the intrinsic limit could be realized in MoS_2_ and WSe_2_ [10]. It has also been reported that the stacking of WSe_2_ and MoSe_2_ MLs enables interlayer excitonic emission that is not present in either WSe_2_ or MoSe_2_ MLs alone [8]. Because numerous stacking orders with various materials are possible, new approaches may lead to interesting physical properties.

However, during the fabrication process of transferring one layer to another, bubbles are formed at the interfaces either intentionally or unintentionally [11,12]. The bubbles, in general, deteriorate the intrinsic material properties of the structure by inducing strain variance and structural defects. On the other hand, it has also been reported that the strain and defects caused by the bubbles lead to enhanced emission intensity and an emission energy shift [13]. These facts reveal the possible usage of bubbles as another external factor to tailor the emission properties of ML TMDs. Despite this potential, the underlying optoelectronic properties of the bubbles existing at the interfaces of TMD heterostructures are not fully understood.

In this paper, we report spatially resolved photoluminescence (PL) and PL excitation (PLE) measurements of a hBN-encapsulated WSe_2_ ML. The PL mapping shows enhanced localized exciton (LX) emission and reduced free exciton (FX) emission at the locations of the bubbles. The PLE results indicate that the absorption probabilities at energies between the n = 1 and n = 2 series of the A-exciton significantly differ at the sites of the bubbles compared to the pristine areas, which can be utilized to improve the bubble imaging in the PL mapping. We also find measurable differences in the PL spectra of the bubbles depending on whether they are positioned at the bottom or top hBN interface.

## 2. Materials and Methods

A hBN-encapsulated WSe_2_ monolayer was obtained by sequentially transferring a bottom hBN flake, ML WSe_2_, and top hBN flake, which were mechanically exfoliated from bulk crystals, onto a quartz substrate [14]. In the PL experiments, an objective lens with a numerical aperture of 0.5 was used to collect PL signals and to focus the laser beam onto the sample with a spot size of about 1 μm. The spot size was measured by both the knife edge method and charge-coupled device (CCD) intensity. The collected emission was sent to a spectrometer (Acton Research Corporation SpectroPr-275) equipped with a thermoelectrically cooled CCD (Andor iDus 401, Abingdon, UK). The sample was kept at a temperature of 80 K inside an optical cryostat (Linkam Scientific, Tadworth, UK), in which motorized motion was used to enable spatially resolved PL mapping. Laser power was in the range 50–300 μW. In the PL excitation measurements, we used a home-built broadband Ti:sapphire laser that covers a wavelength range of 660–980 nm.

## 3. Results and Discussion

**Figure 1:**
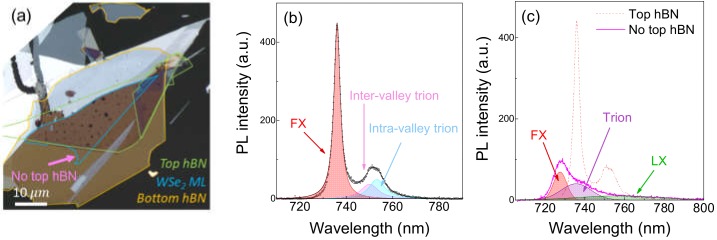
(**a**) Optical microscope image of the WSe_2_ monolayer encapsulated by hBN. (**b**) Photoluminescence spectrum of the WSe_2_ ML in hBN at 80 K. The trion fine structure (pink and cyan lines) contributes to the asymmetric line shape of trion emission. (**c**) PL spectra of WSe_2_ ML with (red dotted line) and without (pink line) capping hBN layers.

Figure 1a shows a microscope image of the fabricated hBN encapsulated WSe_2_ monolayer. In the figure, the top and bottom hBN flakes are shown with green and orange lines, respectively, and the blue line indicates the WSe_2_ ML region. Through hBN encapsulation, it is expected that the crystal quality of the WSe_2_ is enhanced to better exhibit its intrinsic physical properties. The encapsulation, by blocking the direct contaminations of WSe_2_ by ambient molecules, is also favorable in preserving sample quality. However, the image in Figure 1a clearly displays the presence of bubbles in the encapsulated WSe_2_ ML, which were unintentionally formed during the transfer process of WSe_2_ or top hBN. It has been reported that the bubbles are filled with hydrocarbons and impact the physical and electrical properties of the structure including strain variation [13].

Figure 1b shows a PL spectrum from a pristine WSe_2_ ML region in Figure 1a, which is free from bubbles. Here, the excitation wavelength is 532 nm with an average laser power of 50 μW. As indicated by shaded regions, the PL spectrum can be decomposed into three Lorentzian peaks located at 736, 750, and 753 nm. The dominant peak observed at 736 nm has a sharp full width at half maximum linewidth of 7 meV, which corresponds to FX emission. The two lower energy peaks are consistent in terms of energy separation with the trion fine structure reported in [7]. The high-energy peak at 750 nm corresponds to the inter-valley trions consisting of one electron at the K-valley and another at the K’-valley, while the peak at 753 nm corresponds to the intra-valley trions with both electrons in the same valley [4,7,14]. This PL spectrum can provide important information such as the exciton and trion binding energies in studying the optical properties of 2D semiconductors. We find that the PL spectrum exhibits strong local variations in the spectral shape as well as in the signal intensity.

While the PL signal in Figure 1b was obtained from the fully encapsulated hBN–WSe_2_–hBN region, we show in Figure 1c the PL signal from the hBN–WSe_2_ region, as indicated by the pink arrow in Figure 1a, where WSe_2_ is without the top hBN capping layer. The PL at this open region exhibits considerable linewidth broadening, about three times in the case of FX, with respect to the encapsulated site. In addition to the FX and trion, a broad low-energy emission emerges, which results from the LX, as shown with the green line in Figure 1c. Both the linewidth broadening and the LX in the PL signal result in deteriorated optical properties at the open site in comparison to the encapsulated site. Another apparent difference between the two sites is observed in the exciton energy, in which the FX of the open WSe_2_ is 20 meV higher than the FX from the capped WSe_2_. We note that environmental effects strongly influence the exciton binding energies of monolayer TMDs [15,16], and here, the hBN capping induced the observed energy difference of the FX.

**Figure 2:**
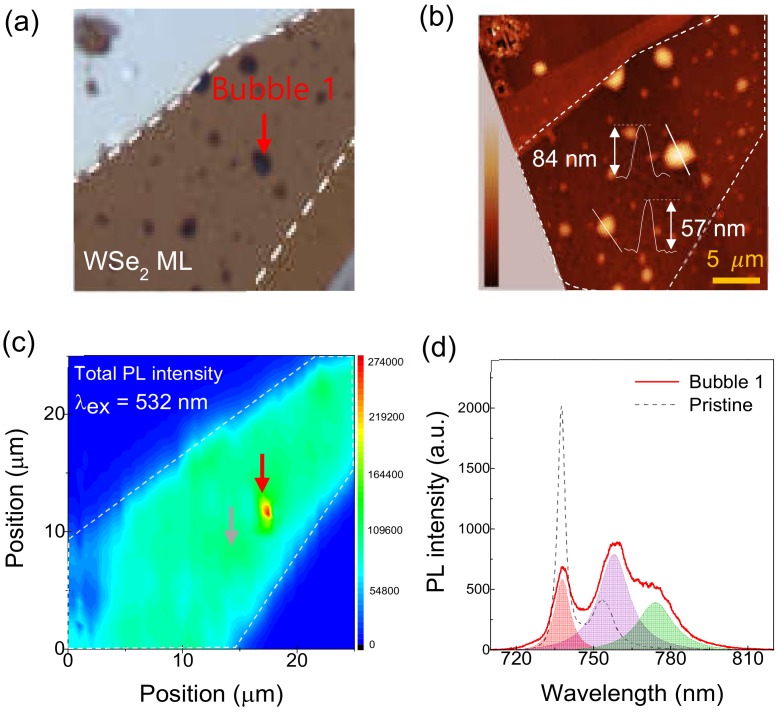
(**a**) Magnified microscope image of the sample in Figure 1a. (**b**) Atomic force microscope image of the same region. (**c**) Spatially resolved PL with an excitation wavelength of 532 nm. Total PL intensity is shown via contour plot from the same region. The red and gray arrows indicate the measured bubble 1 and pristine sites, respectively. (**d**) PL spectra of the bubble and pristine areas shown with solid red and dotted gray lines, respectively.

To gain further insight into the site-sensitive exciton properties, we performed spatially resolved PL measurements. The magnified optical microscope image in Figure 2a and the atomic force microscope (AFM) image in Figure 2b both display several bubble formations. The AFM image indicates that the typical size of the bubbles is a few tens of nanometers in thickness and up to about three micrometers in width. For instance, one large bubble, labeled as bubble 1 in Figure 2a, has a thickness of 84 nm and a diameter of about 2.9 μm.

Interestingly, when the excitation wavelength is 532 nm, the total PL intensity is rather pronounced at bubble 1 as compared to other pristine sites (Figure 2c). Moreover, the PL spectrum at bubble 1 exhibits a drastically different shape from the shape at pristine sites; in Figure 2d, we have fitted the PL spectrum with three Lorentzian peaks located at 738, 758, and 774 nm. The low energy peak at 774 nm, which is not visible at the pristine sites, results from the emission at the LX. The LX is known to be generated at defect sites, such as Se or W vacancies in WSe_2_ ML [17,18]. This kind of enhanced LX emission at the bubbles was also observed in other TMD monolayers like MoS_2_ and WS_2_ [13]. Because the FX can relax into lower energy defect states, the FX emission at bubble 1 is smaller than that at the pristine sites. The 738 and 758 nm PL peaks correspond to FX and trion emission, respectively. The energy separation between the FX and trion peaks is about 44 meV, which is somewhat higher than the generally reported value of about 30 meV of trion binding energy in pristine WSe_2_ ML [4,7]. It has been reported that trion binding energy varies from 21 meV to 50 meV depending on doping [7]. Such a large trion binding energy as found here may represent n-type doping at the bubble site of WSe_2_ ML. According to research from Courtade et al., trion binding energy increases in the n-type doping regime, with trion emission intensity overtaking FX intensity, which is consistent with our result from bubble 1.

**Figure 3:**
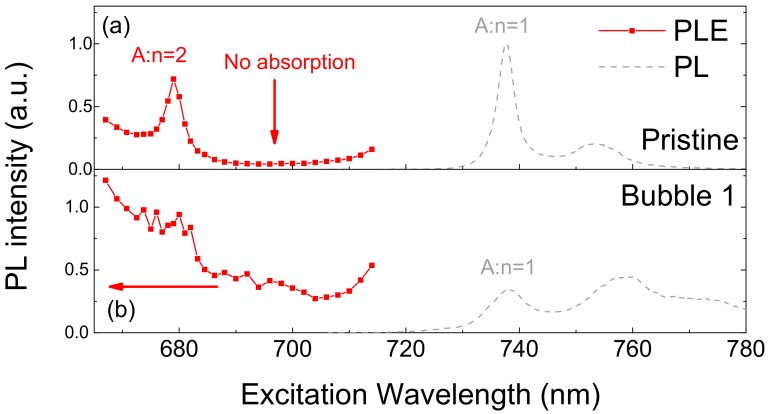
Excitation wavelength-dependent PL intensity at 80 K (red lines), and PL spectra from a pristine area and bubble 1 (dashed gray line). The PLE spectra indicate significant excitation wavelength-dependent emission characteristics for both bubbles and pristine areas in WSe_2_ ML.

While the bubbles can be discerned from their different emission spectra, the bubbles are not disclosed well in the total PL intensity mapping at 532 nm excitation, as in Figure 2c. In order to check the possibility of other excitation conditions as a means for sensitive bubble detection, we performed PLE experiments at a pristine site and bubble 1, with results presented in Figure 3a and Figure 3b, respectively. Because PL intensity is proportional to the number of absorbed photons, PLE spectra provide information on the absorption spectrum. Here, on account of the large exciton binding energy, absorption below the energy gap is determined by a series of Rydberg excitons in the ML WSe_2_. In the pristine area in Figure 3a, we find that the resonance peak at 680 nm corresponds to the n = 2 peak of the A-exciton [18], while the n = 1 peak is located at the FX peak in the PL signal. In principle, no energy states are expected between the n = 1 and n = 2 resonances of the A-exciton, which corresponds to the very small PL intensity at excitation wavelengths around 700 nm, as shown in Figure 3a. In contrast, the n = 2 exciton transition is not clearly observed from the PLE spectrum of bubble 1 in Figure 3b. This phenomenon is possibly due to the substantial broadening of the exciton linewidth, which blurs the n = 2 exciton. Also, in contrast to the pristine site, the PLE intensity at bubble 1 is fairly large between the n = 2 and n = 1 excitons, which is partially explained by the exciton broadening. This non-negligible absorption might also have resulted from defect-state creation at the bubble site. The absorption coefficient in this region, which is sensitive to local properties, can be utilized for the purpose of imaging the crystal quality of ML WSe_2_.

**Figure 4:**
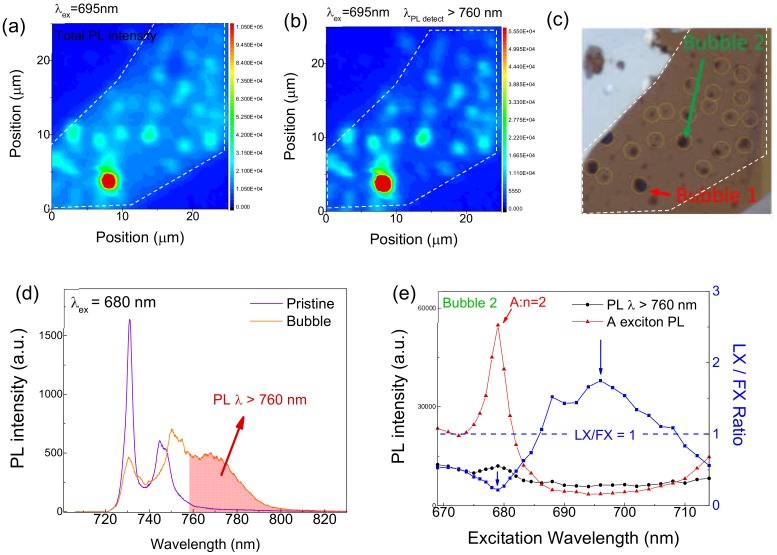
(**a**) Total PL intensity mapping of spatially enhanced LX emission using 695 nm excitation wavelength, and (**b**) PL intensity of λ_detect_ > 760 nm mapping. (**c**) Microscopic image of the mapping area. (**d**) PL spectrum of WSe_2_ ML from bubble 2 and a pristine site using 680 nm excitation wavelength. Area of λ_detect_ > 760 nm is shown. (**e**) Excitation wavelength dependence of FX (red) and LX (black) intensity of WSe_2_ ML for bubble 2. The LX/FX ratio is shown in blue.

With the purpose to better visualize the bubbles through PL intensity, we select the 695 nm wavelength at which the PL intensity at the bubbles is strong but negligible at the pristine sites; Figure 4a shows PL mapping results with 695 nm excitation. Noticeably, in contrast to the PL mapping with 532 nm excitation wavelength in Figure 2c, the bubbles in Figure 4a are clearly observed due to wavelength-dependent selectivity. Though the bubbles are discernable from the total PL intensity imaging in Figure 4a, we get even better visibility by integrating the LX signals selectively. Figure 4b is the result of applying an integration window with a short cutoff wavelength of 760 nm, as marked by the shaded area in Figure 4d. This enhanced visibility is attributed to the combined effects of reduced LX emission and negligible excitation probability at the pristine sites. The location of the bubbles from the PL mapping matches with the optical microscope image in Figure 4c. Figure 4e shows the excitation wavelength dependence of the FX (red) and LX (black) emission at bubble 2. Here, we choose bubble 2 instead of bubble 1 because the PL intensity at bubble 2 is closer to the other bubbles within the sample. The emission ratio of LX relative to FX (LX/FX) is also shown in Figure 4e; since each individual bubble shows drastically different PL spectra, the FX and LX emission are distinguished by the 760 nm cutoff wavelength. This PLE spectra detecting a different wavelength range informs the presence of a dominant defect generation channel at energy levels between A:n = 1 and A:n = 2. The dominant LX emission is even more clearly observed through the LX/FX ratio (blue line in Figure 4e). While an excitation laser wavelength of 680 nm shows the lowest LX/FX ratio for bubble 2, bubble imaging at this wavelength is not as clear as the imaging acquired from PL mapping with a 695 nm excitation laser wavelength. Our observation indicates that LX and FX can be selectively generated depending on excitation laser wavelength. It is also noted that FX peak shifts at the bubbles are not noticeable in our experiment, as shown in Figure 4d. In addition to locally enhanced LX emission at the bubbles, each bubble shows a different PL intensity, for example bubbles 1 and 2 in Figure 4a.

**Figure 5:**
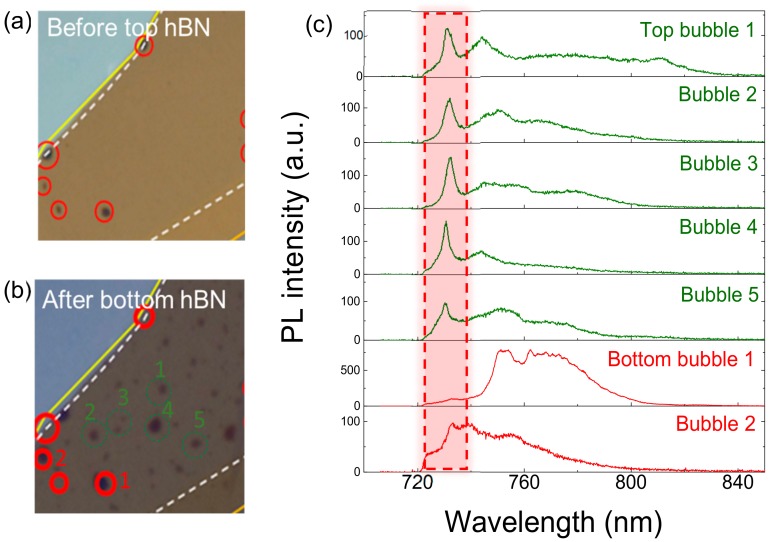
Microscope image of WSe_2_ ML on bottom hBN: (**a**) before top hBN transfer, and (**b**) after top hBN transfer. Bottom and top bubbles are circled in red and green, respectively. (**c**) PL spectra of WSe_2_ ML on corresponding bubbles labeled in (**b**).

Bubbles can be generated in each step of the hBN or WSe_2_ transfer process. Because of the rather large thickness of the bottom hBN, there is little chance of bubble formation during the bottom hBN transfer. Whether a bubble is located at the bottom-hBN–WSe_2_ interface or the WSe_2_–top-hBN interface can be determined by comparing microscope images captured during sample fabrication. The microscope image in Figure 5a taken just after WSe_2_ transfer shows several bubbles formed at the bottom interface of the WSe_2_ layer. On the other hand, the image in Figure 5b taken after top hBN transfer indicates additional bubbles at the top interface, was created during the top hBN transfer. In Figure 5b, we mark a number of bottom and top bubbles with red and green circles, respectively. Figure 5c shows the corresponding PL spectra at the bubbles designated in Figure 5b. While the LX emission is enhanced at both top and bottom bubbles, there is a tendency for FX emissions at the bottom bubbles to rather severely reduce, compared to FX emissions at the top bubbles. This behavior possibly results from the different morphology of the WSe_2_ layer between the two cases. While the WSe_2_ layer will be flat at the locations of the top bubbles, the bottom bubbles will force the WSe_2_ layer to form around the bubble shape, which will generate strain and/or crystalline defects near the bottom bubbles. As compared to the other measured bubbles in Figure 5c, the PL intensity at bottom bubble 1 is exceptionally high, being even stronger than at pristine sites. At this moment, we do not understand the reason for this strong inter-defect variation of the emission efficiency, but we expect that further investigations on defect types or defect-induced emission mechanisms will help solve this issue.

## 4. Conclusions

In conclusion, spatially resolved PL measurements show a position-dependent large variation of PL intensity even in high quality WSe_2_ monolayer encapsulated with hBN. Optical microscope images and AFM scanning results confirm the presence and dimension of bubbles at both bottom and top interfaces of the WSe_2_ monolayer, at which strong localized exciton emissions are observed below the free exciton energy. PLE spectra comparing bubble and pristine sites indicated a large contrast ratio at energies between the n = 1 and n = 2 excitons, where no transition probability is expected in an ideal system. By choosing an excitation wavelength of 695 nm, which satisfies this condition, we could clearly visualize images of the bubbles from PL mapping. Even clearer bubble imaging was enabled by limiting the detection wavelength into the LX emission range. We also found that the bubbles, depending on whether they are located at the bottom or top interface of the WSe_2_ layer, can exhibit different characteristics regarding emission properties or related strain effects. This study demonstrates the importance of choosing appropriate excitation wavelengths when performing spatially-resolved PL imaging, a finding applicable to other optoelectronic materials including monolayer TMDs.
